# TENS versus foam rolling for recovery after eccentric exercise–induced muscle damage in elite female volleyball players: an exploratory randomized controlled trial

**DOI:** 10.1186/s13102-026-01782-x

**Published:** 2026-06-03

**Authors:** Shahla Shahnavazi, Mohammadreza Rezaeipour, Ahmad Mir

**Affiliations:** 1 Department of Sport Sciences, University of Sistan and Baluchestan, Zahedan, Iran; 2 General Directorate of Sports and Youth of Sistan and Baluchestan Province, Zahedan, Iran

**Keywords:** Creatine kinase, Delayed-onset muscle soreness, Eccentric exercise, Elite athletes, Foam rolling, Muscle damage recovery, Neuromuscular performance, Self-myofascial release, Transcutaneous electrical nerve stimulation, Volleyball

## Abstract

**Background:**

Delayed-onset muscle soreness (DOMS) compromises performance and prolongs recovery in elite athletes. Transcutaneous electrical nerve stimulation (TENS) and foam rolling (FR) are widely used but have never been directly compared in elite female volleyball players. This study compared the effects of these two supplements on biochemical and functional recovery following eccentric exercise-induced muscle damage in elite female volleyball players.

**Methods:**

As part of this exploratory pilot study, thirty elite female volleyball players (age 23.4 ± 2.8 years; height 178.6 ± 5.4 cm; body mass 68.2 ± 6.1 kg; training experience 8.3 ± 2.1 years in Iran’s Premier League) were randomly assigned 1:1:1 to TENS (*n* = 10), FR (*n* = 10), or control (CON; *n* = 10) groups using concealed computer-generated allocation. DOMS was induced by five sets of 15 eccentric leg-press repetitions at 110% concentric one repetition maximum. TENS (120–150 Hz, 100 µs, 10–30 mA, 20 min) was applied to the dominant quadriceps and hamstrings. FR (20 min) targeted the quadriceps, hamstrings, iliotibial band, gastrocnemius, and gluteals muscles. Serum creatine kinase (CK), vertical jump height, and anaerobic peak power (Running based Anaerobic Sprint Test) were measured at baseline and 1, 24, and 48 h postexercise. Mixed-design repeated-measures ANOVA with Bonferroni-adjusted pairwise comparisons was used.

**Results:**

CK showed a significant Group × Time interaction (*p* < 0.001, η²*p* = 0.24). TENS lowered CK vs. CON at 1 h (− 186 U/L, 95% CI − 289 to − 83; *p* = 0.001, d = 1.98), 24 h (− 312 U/L, 95% CI − 442 to − 182; *p* = 0.001, d = 2.32), and 48 h. FR lowered CK vs. CON only at 48 h (− 166 U/L, 95% CI − 273 to − 59; *p* = 0.003, d = 1.32). Vertical jump recovery at 48 h was superior in both interventions vs. CON (TENS + 4.8 cm, 95% CI 2.1–7.5, *p* = 0.001, d = 1.11; FR + 3.7 cm, 95% CI 1.4–6.4, *p* = 0.001, d = 0.85), reaching 96.3% and 95.7% of baseline vs. 85.8% in CON. No group differences were observed for anaerobic power (Group × Time *p* = 0.086, η²*p* = 0.08). Muscle soreness was lower in both active groups than in the CON group at 48 h (*p* < 0.01, d ≥ 1.48).

**Conclusions:**

In this pilot cohort, TENS and FR improved recovery relative to control with different CK temporal patterns but comparable vertical jump restoration at 48 h. However, given the exploratory nature of the study, these results should be interpreted as preliminary. TENS had earlier CK reductions, relevant for 24-h recovery windows; however, both produced comparable functional recovery by 48 h, and neither showed consistent superiority across outcomes. FR offers a practical, cost-effective alternative to achieve equivalent functional restoration within 48 h.

**Trial registration:**

ClinicalTrials.gov, NCT07438197, registered on 22/02/2026. Retrospectively registered.

**Graphical Abstract:**

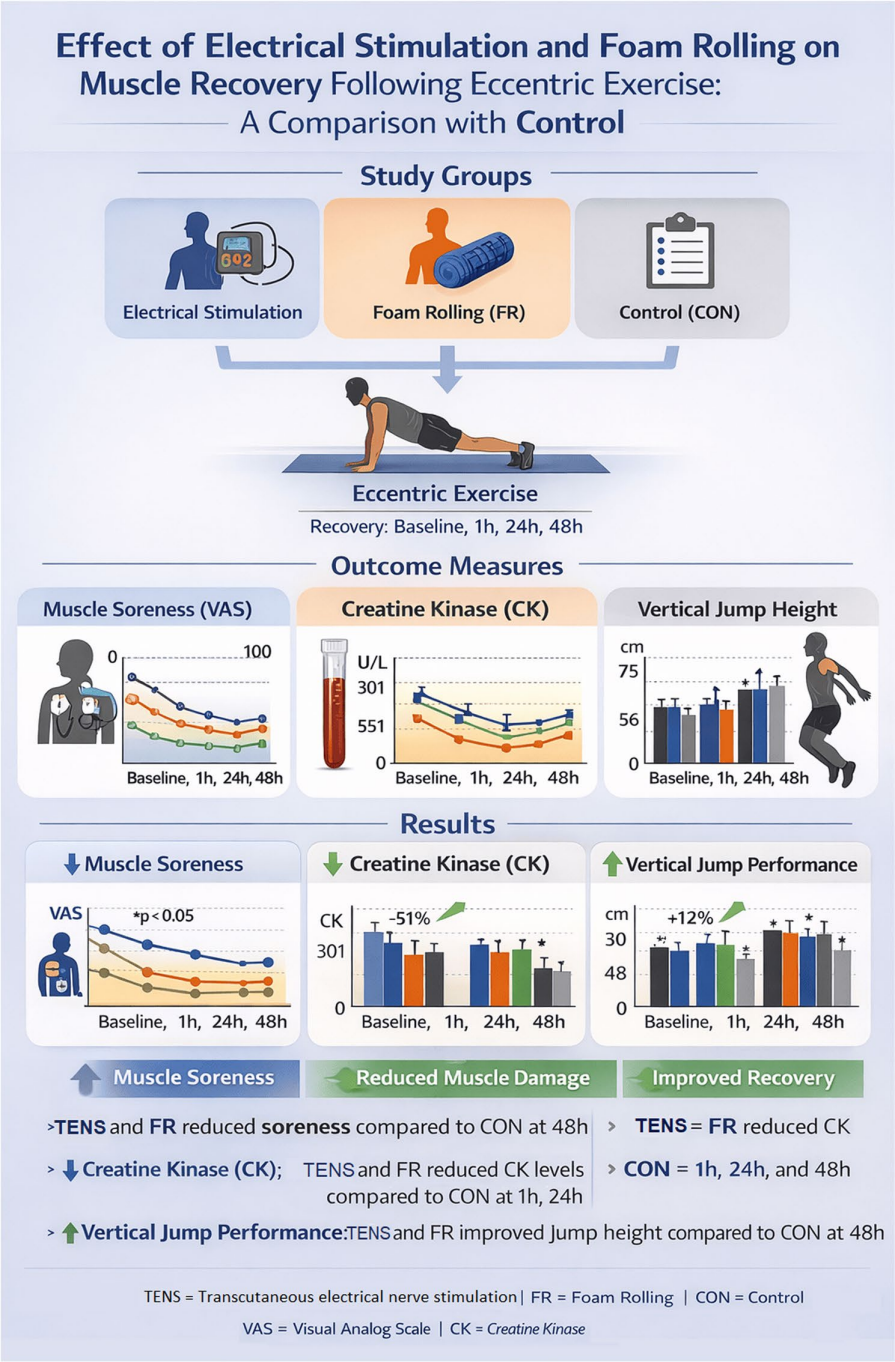

**Supplementary Information:**

The online version contains supplementary material available at 10.1186/s13102-026-01782-x.

## Plain Language

What was already known

Intense exercise, particularly movements that involve muscle lengthening, such as landing from jumps, often causes delayed muscle soreness and temporary reduction in strength. Two recovery methods — electrical nerve stimulation applied through the skin and foam rolling — are commonly used by athletes to reduce soreness and support a faster return to training. However, no previous study has directly compared these approaches in elite female volleyball players, leaving coaches and sports medicine practitioners without clear evidence to guide their method selection.

What this study found

Thirty elite female volleyball players from Iran’s Premier League completed a demanding lower-body exercise protocol designed to induce muscle soreness. Participants were assigned to receive transcutaneous electrical nerve stimulation, foam rolling, or passive rest. Blood samples and physical performance tests were collected before exercise and at one, twenty-four, and forty-eight hours afterward.

Both active recovery methods produced better outcomes than passive rest. Transcutaneous electrical nerve stimulation reduced a blood marker of muscle damage more rapidly — within one hour — whereas foam rolling achieved comparable reductions by forty-eight hours. Both methods enabled players to recover nearly all of their jumping ability within forty-eight hours (approximately 96% of pre-exercise performance), compared with 86% recovery in the passive rest group.

Why it matters

When athletes are required to compete or train again within twenty-four hours, transcutaneous electrical nerve stimulation may provide faster recovery of muscle integrity. Foam rolling remains a practical, low-cost alternative that produces comparable functional recovery within two days. Coaches and practitioners can use these findings to select recovery strategies based on the time available between competitions.

**Supplementary Information:**

The online version contains supplementary material available at 10.1186/s13102-026-01782-x.

## Key points

1. No previous study has directly compared transcutaneous electrical nerve stimulation and foam rolling for recovery from exercise-induced muscle damage in elite female volleyball players.

2. TENS lowered serum creatine kinase within one hour, whereas foam rolling reached significance only at forty-eight hours — indicating distinct biochemical recovery timelines.

3. Despite these biochemical differences, both modalities restored vertical jump height to approximately 96 % of baseline by forty-eight hours versus 86 % with passive rest.

4. These findings indicate that transcutaneous electrical nerve stimulation may be prioritized when rapid biochemical recovery is required between closely scheduled competitions, whereas foam rolling provides a portable, self-administered alternative that achieves equivalent functional recovery at forty-eight hours.

**Supplementary Information:**

The online version contains supplementary material available at 10.1186/s13102-026-01782-x.

## Introduction

Delayed-onset muscle soreness (DOMS) extends beyond simple residual stiffness following a demanding training session. This reflects structural disruption of skeletal muscle fibers, which can interfere with training periodization and compromise competitive readiness [1]. These implications are particularly significant for elite volleyball players, whose performance depends on explosive vertical jumping and sustained lower-body power; even moderate residual soreness can measurably reduce on-court performance capacity [2]. DOMS typically develops 12–72 h after unaccustomed eccentric exercise and manifests as localized pain, swelling, and functional impairment [3]. At the cellular level, eccentric loading disrupts sarcomere architecture, initiating inflammatory cascades and releasing intracellular proteins into the bloodstream [4]. Among these proteins, creatine kinase (CK) is widely used as a circulating biomarker to quantify the severity of exercise-induced muscle membrane disruption [5].

Recovery strategies span a broad continuum and include passive rest, stretching, massage, hydrotherapy, and electrical modalities [6]. However, meta-analytic evidence indicates that traditional static and proprioceptive neuromuscular facilitation (PNF) stretching protocols provide limited relief from DOMS symptoms [7], shifting attention toward modalities supported by emerging mechanistic evidence. Transcutaneous electrical nerve stimulation (TENS) has documented neurophysiological mechanisms related to large-diameter afferent fiber activation and local microcirculatory enhancement [8, 9], whereas foam rolling (FR) is supported by growing evidence of mechanical tissue compression, improved fascial compliance, and autonomic modulation [10, 11]. Both modalities have gained prominence partly because they can be self-administered with minimal equipment. TENS enhances local blood flow and preferentially activates large-diameter sensory fibers, mechanisms that may accelerate metabolite clearance and modulate pain perception through the gate-control pathway [8, 9]. Electrically induced muscle contractions have also been proposed to facilitate lymphatic drainage and reduce interstitial edema [12]. In contrast, FR applies bodyweight-driven compressive and shear forces using a cylindrical device, with reported benefits including improved tissue compliance, reduced fascial restrictions, and enhanced parasympathetic activity [10]. Beyond manual modalities, non-pharmacological interventions are increasingly recognized for their ability to influence recovery through vascular and neuromuscular pathways. For instance, recent evidence suggests that neuromuscular electrical stimulation can produce measurable functional and exercise capacity adaptations, supporting the physiological plausibility of stimulation-based interventions beyond simple pain modulation [13]. Similarly, vascular-targeted strategies such as ischemic preconditioning have demonstrated the potential to delay neuromuscular fatigue development in athletic populations, likely through integrated vascular and neuromuscular pathways [14]. Positioned within this broader framework, TENS may offer a unique synergy of sensory and microcirculatory benefits that justify its investigation as a post-exercise recovery tool. A systematic review and meta-analysis demonstrated that FR can positively influence both acute performance and recovery, although the effect magnitudes vary depending on the rolling duration, pressure, and timing [11]. Notably, postexercise FR appears to attenuate DOMS symptoms and helps preserve muscle function [15, 16].

Despite growing interest in these modalities, direct head-to-head comparisons remain limited [17]. Most investigations have evaluated individual interventions against passive control conditions, providing practitioners with little guidance when selecting between competing recovery strategies [18]. This limitation is compounded by the frequent reliance on recreational populations, whose recovery physiology differs substantially from that of elite competitive athletes [19]. Volleyball offers a particularly informative model because competitive match play may involve more than 100 high-intensity vertical jumps combined with rapid lateral movements and overhead striking [20]. Although much of the available workload data derive from elite male cohorts, comparable eccentric demands have been documented in elite female competition, where repeated jump-landing cycles impose substantial lower-extremity loading. Therefore, the quadriceps, hamstrings, and gastrocnemius muscles experience repeated eccentric loading during the landing phases, increasing the susceptibility to DOMS in volleyball athletes [21].

To date, no study has directly compared the effects of TENS and FR in elite female volleyball players, a population whose sport-specific eccentric demands increase their vulnerability to lower-extremity muscle damage [22, 23]. Existing literature examining electrical modalities in volleyball has not included such direct comparisons [24, 25], and findings related to FR and CK responses remain inconsistent. Individual trials report inconsistent CK responses to FR: Pearcey et al. [26] demonstrated significant reductions, whereas D’Amico and Gillis [27] detected no measurable effect. These discrepancies may partially reflect differences in rolling protocols and their implementation [19]. Consequently, practitioners lack clear evidence to guide the selection of the two widely accessible recovery tools.

Therefore, this study compared the effects of TENS, FR, and passive recovery on serum CK concentrations, vertical jump performance, and anaerobic power following a standardized eccentric exercise protocol in this population. We hypothesized that both active interventions would outperform passive rest in attenuating markers of muscle damage and preserving neuromuscular function, with TENS potentially producing earlier benefits because of its established vascular and sensory neurophysiological mechanisms.

## Materials and methods

### Study design

This three-arm, parallel-group exploratory pilot randomized controlled trial was conducted between September and December 2020 at the Zahedan Olympic Village in Iran. Participants were randomly allocated in equal proportions to the intervention or control groups using a computer-generated sequence. Allocation was concealed in sequentially numbered, opaque, sealed envelopes prepared by a researcher not involved in enrollment or assessment. Because of the nature of the interventions, participant blinding was not feasible; however, outcome assessment and data analysis were performed by investigators blinded to group allocation. This study adhered to the CONSORT 2010 guidelines for reporting randomized controlled trials.

### Ethical approval

The study protocol was approved by the Ethics Committee of the University of Sistan and Baluchestan (No. IR.USB.REC.1399.007). All participants provided written informed consent prior to enrollment, and all procedures adhered to the principles of the Declaration of Helsinki.

### Participants

Elite female volleyball players competing in Iran’s Premier League were recruited through team coaches and sports medicine staff. According to the participant classification framework proposed by McKay et al. [28], these athletes correspond to Tier 4 (elite/international-level), reflecting sustained participation in the highest domestic competitive league with full-time training commitments. Of the 38 athletes initially screened, eight were excluded: four reported musculoskeletal injuries within the preceding six months, two were taking anti-inflammatory supplements, and two had performed lower-body strength training within 72 h of the scheduled testing session.

Inclusion criteria were as follows: age 18–28 years; at least five years of competitive volleyball experience; current participation in the national Premier League; training frequency of at least five sessions per week; and absence of musculoskeletal injury during the preceding six months. Exclusion criteria were as follows: use of anti-inflammatory medications or supplements; contraindications to TENS (cardiac pacemaker, epilepsy, pregnancy); exposure to the study interventions within the preceding four weeks; and lower-body strength training within 72 h prior to testing.

Thirty athletes met all eligibility criteria and completed all scheduled sessions (age, 23.4 ± 2.8 years; height, 178.6 ± 5.4 cm; body mass, 68.2 ± 6.1 kg; training experience, 8.3 ± 2.1 years). Menstrual cycle phase was not standardized across participants; however, each individual completed the full testing sequence within a 48-hour window, thereby minimizing intra-individual hormonal variation across time points. All participants completed familiarization sessions for the Sargent jump and Running-based Anaerobic Sprint Test (RAST) protocols 5–7 days before baseline testing to reduce learning effects. Sample size adequacy is addressed in the Statistical Analysis section.

### Randomization and group allocation

The random allocation sequence was generated using Random Allocation Software (version 2.0; Isfahan University of Medical Sciences, Iran). Simple randomization without blocking or stratification was used. In a sample of this size, this approach increases the theoretical risk of residual baseline imbalance despite non-significant omnibus comparisons. Although baseline characteristics were statistically equivalent across groups (Table 1; all *p* > 0.40), the possibility that subtle, undetected differences influenced outcomes cannot be excluded. Stratification by baseline performance level or 1RM would have provided additional protection against confounding and is recommended for future trials of this scale. An independent researcher not involved in the recruitment or testing prepared the allocation list. Group assignments were placed in sequentially numbered, opaque, sealed envelopes to ensure concealment until baseline assessments were completed. This procedure resulted in three groups of ten participants each: transcutaneous electrical nerve stimulation (TENS; *n* = 10), foam rolling (FR; *n* = 10), and control (CON; *n* = 10). Baseline group characteristics are presented in Table 1.

**Table 1:** Baseline participant characteristics by experimental group

Variable	TENS (*n* = 10)	FR (*n* = 10)	CON (*n* = 10)	*p*-value
Age (years)	23.1 ± 2.5	23.8 ± 3.1	23.2 ± 2.9	0.842
Height (cm)	179.2 ± 5.8	177.8 ± 4.9	178.9 ± 5.7	0.816
Body mass (kg)	67.8 ± 5.9	69.1 ± 6.8	67.6 ± 5.4	0.834
BMI (kg·m⁻²)	21.1 ± 1.4	21.9 ± 1.8	21.1 ± 1.5	0.447
Training experience (years)	8.5 ± 2.3	8.1 ± 1.9	8.2 ± 2.2	0.912

Values are presented as mean ± standard deviation. *p*-values were derived from one-way ANOVA

*BMI* Body mass index, *CON* Control group, *FR* Foam rolling, *TENS* Transcutaneous electrical nerve stimulation

### Procedures

#### One-repetition maximum testing

Concentric one-repetition maximum (1RM) for the 45° angled leg press (Model LP-45; Pars Varzesh Negin [Inpars], Tehran, Iran) was determined for each participant seven days before the experimental session, following established guidelines for maximal strength assessment [29]. Following a standardized warm-up consisting of 10 repetitions at approximately 50% of the estimated 1RM, loads were increased in 5–10% increments until the participant was unable to complete a repetition through the full range of motion [27]. A maximum of five attempts were permitted, with 3–5 min of rest between attempts. The highest successfully completed load was recorded as the 1RM and used to prescribe the eccentric exercise intensity.

### DOMS induction protocol

All participants completed the eccentric exercise protocol between 08:00 and 10:00 under standardized laboratory conditions (ambient temperature: 22 ± 1 °C). A standardized warm-up preceded the protocol and included 10 min of stationary cycling at 60 W on an electronically braked ergometer (Monark 828E; Vansbro, Sweden), followed by 5 min of dynamic stretching.

The eccentric protocol consisted of five sets of 15 eccentric-focused repetitions on the same 45° angled leg press, with a load fixed at 110% of concentric 1RM. A 4-second controlled eccentric phase was paced by a digital metronome application (Metronome Beats; Stonekick, UK) delivered through external speakers. Concentric return of the load was performed by two trained research assistants. Ninety seconds of rest was provided between sets.

This protocol reliably induces DOMS that peaks within 24–48 h, making it suitable for examining short-term recovery responses under controlled conditions [30–32]. Participants were instructed to maintain habitual dietary intake, hydration practices, and sleep routines throughout the 48-h follow-up period and to abstain from analgesic or anti-inflammatory medications, alcohol, caffeine, and any recovery modalities outside the study protocol. Compliance was monitored using daily self-report checklists documenting sleep duration, fluid intake consistency, dietary adherence, and absence of additional recovery strategies. Nutritional intake was not formally standardized or quantitatively recorded, which may have contributed to inter-individual variability in CK responses. As noted in the Participants section, menstrual cycle phase was neither standardized nor recorded, representing a potential physiological confounder that warrants attention in future female-specific investigations.

### Recovery interventions

Interventions began 30 min after completion of the eccentric protocol and were repeated 24 h later, resulting in two intervention sessions. All the transcutaneous electrical nerve stimulation sessions were administered and supervised by a licensed physiotherapist with at least three years of clinical electrotherapy experience. A trained research assistant supervised the FR and CON sessions to ensure adherence to the protocols. Participants were monitored for adverse events, including skin irritation at the electrode sites, excessive soreness, or vasovagal responses during blood collection. A standardized adverse event form was filled out after each session.

### Transcutaneous electrical nerve stimulation protocol

TENS was applied to the quadriceps and hamstrings of the dominant leg using a clinical-grade stimulator (SL400; Berjis Medical Equipment Co., Tehran, Iran). Four self-adhesive electrodes (5 × 9 cm; Avin Teb, Tehran, Iran) were placed on skin cleaned with a 70% isopropyl alcohol solution. Two electrodes were positioned longitudinally over the rectus femoris muscle, with the proximal electrode approximately 10 cm distal to the anterior superior iliac spine and an interelectrode distance of 5 cm. Two additional electrodes were placed over the biceps femoris, midway between the ischial tuberosity and popliteal fossa. Participants remained supine throughout each session.

Stimulation was applied unilaterally to the dominant leg because of practical constraints related to electrode placement and the capacity of a single device. Because the eccentric protocol loaded both limbs and all functional assessments (Sargent jump and RAST) required bilateral force production, any local physiological benefit from unilateral TENS would be partially diluted in whole-body performance outcomes. This design feature yields a conservative estimate of potential limb-specific effects but simultaneously limits the attribution of observed functional improvements directly to localized stimulation. Future studies should employ bilateral TENS application or limb-specific performance assessments to resolve this issue. However, the eccentric protocol loaded both limbs, and functional assessments (Sargent jump and RAST) required bilateral effort. This asymmetry represents a conservative test of TENS efficacy because any systemic benefit from unilateral stimulation would be diluted in the bilateral outcome measures. Stimulation parameters were selected based on established recommendations for high-frequency sensory-level TENS [8, 9, 12]: biphasic symmetric waveform; frequency 120–150 Hz (continuous high-frequency mode); pulse width 100 µs; intensity 10–30 mA, individually adjusted to produce strong, comfortable paresthesia without visible muscle contraction; duration 20 min per session.

### Foam rolling protocol

Participants used a medium-density, smooth-surface foam roller (15 cm diameter × 45 cm length; Denafoam, Tehran, Iran) and followed a standardized bilateral sequence informed by current best-practice recommendations [15, 16, 19], targeting the quadriceps (anterior thigh), hamstrings (posterior thigh), iliotibial band (lateral thigh), gastrocnemius–soleus complex (posterior lower leg), and gluteal muscles. Each muscle group was rolled for 45 s per side. All five regions were treated bilaterally; however, because the iliotibial band and gluteal muscles cannot be rolled on both sides simultaneously, they were addressed with distinct left- and right-side bouts. Cadence was standardized using verbal pacing cues from the supervising researcher, targeting a rolling speed of approximately 3 cm·s⁻¹. Participants supported their body weight on the roller without applying additional manual pressure. Rolling pressure was not instrumented or externally quantified; consequently, inter-individual variations in body mass, limb geometry, and weight distribution across the roller may have introduced variability in the effective mechanical dose delivered to each muscle group. The rest intervals between muscle groups did not exceed 15 s. Three complete cycles were performed, totaling approximately 20 min per session. When a tender point was encountered, participants paused for 2–3 s before continuing.

### Control condition

Participants in the control group rested quietly in the supine position for 20 min without receiving any therapeutic intervention.

### Outcome measures

All assessments were conducted at baseline (pre-exercise) and at 1, 24, and 48 h postexercise in a fixed sequence: (1) venous blood sampling, (2) visual analog scale assessment, (3) Sargent jump testing, and (4) the Running-based Anaerobic Sprint Test (RAST), with a 5-minute interval between tests. The visual analog scale (VAS) served as a secondary outcome to confirm successful DOMS induction and contextualize functional recovery; it was not designated as a primary endpoint. A single trained assessor, blinded to group allocation, conducted all functional measurements.

### Serum creatine kinase

Venous blood samples (5 mL) were collected from the antecubital vein into serum separation tubes containing a clot activator and a gel separator (Farzaneh Arman Research and Production Center [Fartest], Tehran, Iran). The samples were allowed to clot at room temperature for 30 min and then centrifuged at 1500 × g for 10 min. Serum was stored at − 80 °C until analysis. Creatine kinase (CK) activity was measured using an enzymatic colorimetric assay (CK-NAC, IFCC kinetic method; Darman Faraz Kave, Iran; catalog [CODE,108]) on an automated analyzer (NMABC99200; Nano Mabna Iranian Co., Tehran, Iran), which was calibrated daily according to the manufacturer’s specifications. All samples were analyzed in a single batch, and intra-assay coefficient of variation was 2.1%.

### Vertical jump performance

Explosive lower-extremity power was assessed using the Sargent jump test. Participants stood adjacent to a wall-mounted measuring board graduated in 0.5-cm increments and marked their standing reach height with their dominant hand. They then performed a countermovement jump with an arm swing and touched the board at the peak height. The best of three trials, separated by 30 s of rest, was recorded. Jump height was calculated as the difference between standing reach and peak jump height. This protocol demonstrates high test–retest reliability in athletic populations (intraclass correlation coefficient = 0.93–0.98) [33].

### Anaerobic power

Peak power, mean power, and fatigue index were derived from the RAST (detailed descriptive statistics for mean power and fatigue index are provided in Supplementary Table S1). Participants completed six maximal 35-meter sprints with 10-second passive recovery intervals. Sprint times were recorded using dual-beam infrared timing gates (Witty System; Microgate Srl, Bolzano, Italy; accuracy ± 0.001 s) positioned at hip height at the start and finish lines. Power output was calculated using the following formula: body mass × distance² / time³ [34]. The RAST correlates strongly with Wingate-derived peak power (*r* = 0.87) and has been validated in court-based athletic populations [35].

### Muscle soreness

Perceived soreness in the quadriceps was quantified during a standardized bodyweight squat (feet shoulder-width apart, descent to 90° knee flexion) using a 100-mm VAS anchored at “no soreness” (0 mm) and “worst possible soreness” (100 mm). This dynamic assessment has been validated for eccentric exercise–induced muscle pain [36].

### Statistical analysis

Data were analyzed using SPSS software (version 27; IBM Corporation, Armonk, NY, USA). Normality was assessed using the Shapiro–Wilk test. All variables, including CK concentrations, satisfied the normality assumptions at each time point (all *p* > 0.05); therefore, untransformed data were analyzed. Homogeneity of variance was verified using Levene’s test. Baseline characteristics were compared using one-way analysis of variance (ANOVA).

Primary analyses employed mixed-design repeated-measures ANOVA with Group (TENS, FR, CON) as the between-subject factor and Time (baseline, 1, 24, and 48 h) as the within-subject factor. Greenhouse–Geisser corrections were applied when sphericity assumptions were violated (Mauchly’s test, ε reported). When significant interactions were observed, simple effects comparisons were conducted at each time point using estimated marginal means with the pooled within-cell error term from the omnibus model. Bonferroni adjustments were applied to account for multiple pairwise comparisons at each time-point. No adjustment of the alpha level was made across outcome domains because each domain addressed a distinct mechanistic question (biochemical integrity, neuromuscular function, and subjective recovery). Nevertheless, the examination of multiple outcomes increases the cumulative probability of type I error, and isolated significant findings should be interpreted within this context.

Effect sizes were reported as partial eta squared (η²p) for omnibus effects (small ≥ 0.01, medium ≥ 0.06, large ≥ 0.14) and as Cohen’s d for pairwise comparisons, calculated as the mean difference divided by the pooled standard deviation of the two compared groups, accompanied by 95% confidence intervals. Statistical significance was set at *p* < 0.05.

A prospective sample size calculation based on a predefined primary outcome was not performed because effect size estimates specific to elite female volleyball players undergoing this combined biochemical and performance protocol were unavailable at the time of the study design. The sample of 30 participants was based on feasibility considerations within the accessible elite population. To retrospectively evaluate the adequacy of this sample, a sensitivity analysis was conducted using G*Power version 3.1.9.7 (F-tests, ANOVA: repeated measures, within–between interaction). With *N* = 30, α = 0.05, statistical power = 0.80, three groups, four measurement points, a correlation among repeated measures of ρ = 0.65 (derived from the observed correlation matrix), and a nonsphericity correction of ε = 0.89, the minimum detectable effect size was f = 0.28 (η²*p* ≈ 0.07). Because the observed interaction effect for peak power (f = 0.30, η²*p* = 0.08) exceeded this threshold, the sample size was sufficient to detect effects of this magnitude. Consequently, null findings for anaerobic variables are unlikely to be solely attributable to insufficient statistical power.

## Results

**Fig. 1:**
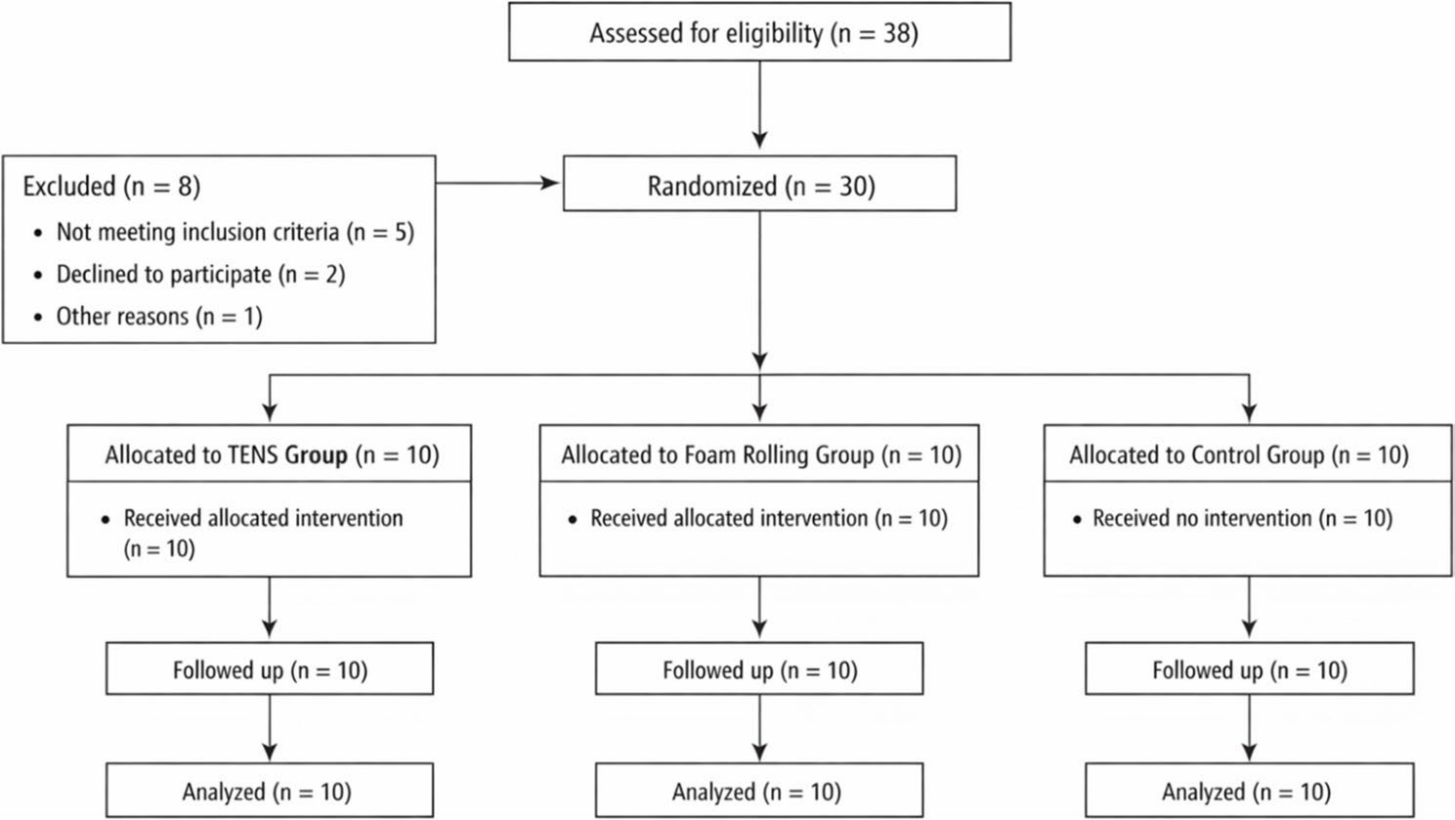
CONSORT flow diagram

All randomized participants (*n* = 30) completed the follow-up and were included in the primary analyses. No participants were lost to follow-up, and no data were excluded from the study. Participant flow through the trial is presented in Fig. 1.

**Fig. 2:**
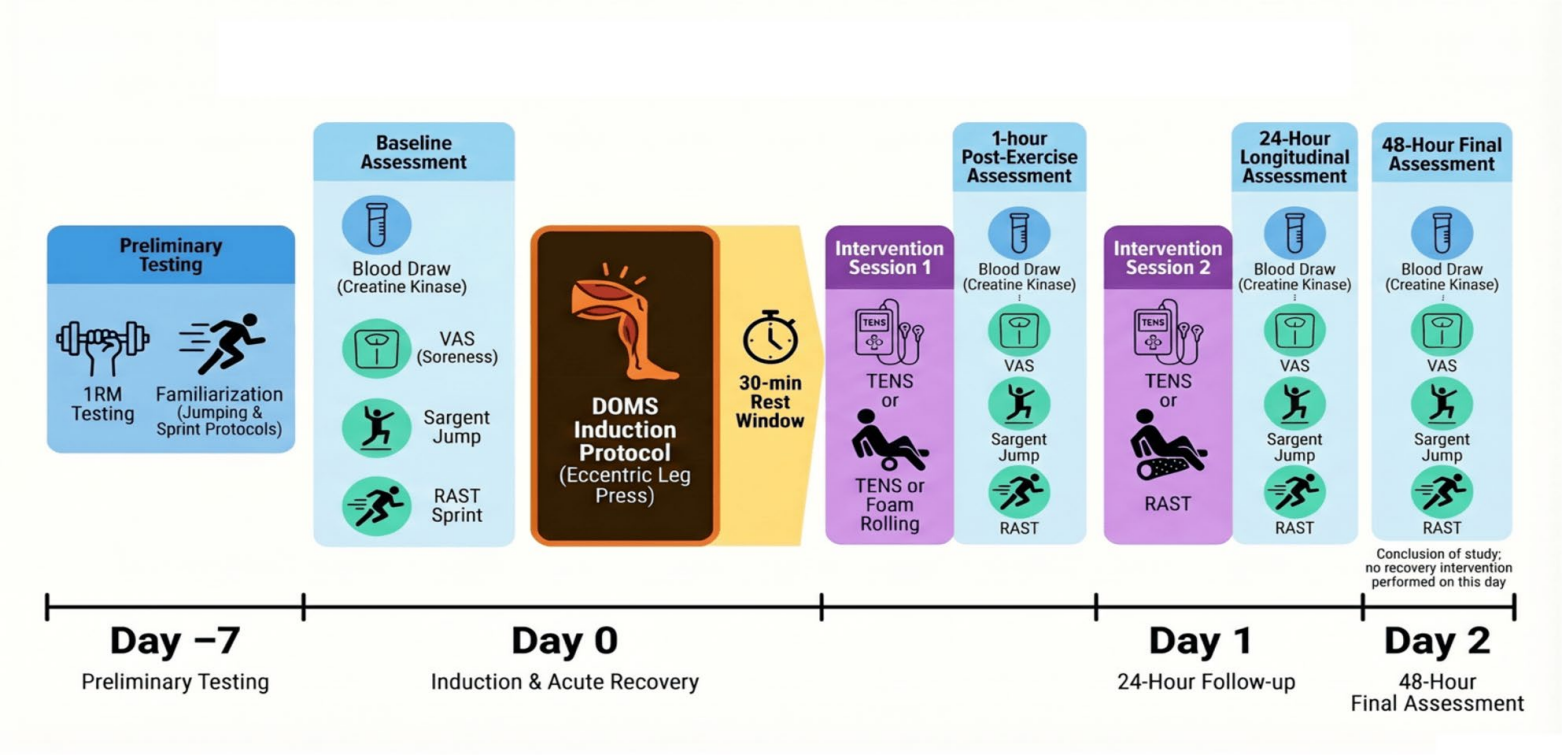
Experimental timeline schematic of the study protocol. Caption: The experimental timeline illustrates the sequence and timing of all study procedures across the trial period, including familiarization and one-repetition maximum testing (Day − 7), baseline assessment and DOMS induction followed by recovery interventions and the 1-h postexercise assessment (Day 0), the second intervention session and 24-h assessment (Day 1), and the final 48-h assessment without intervention (Day 2). At each postexercise time point, assessments were conducted in a fixed sequence: venous blood sampling, visual analog scale rating, Sargent jump test, and Running-based Anaerobic Sprint Test, with 5-min intervals between tests. Legend: Shaded blocks represent the intervention sessions (TENS, FR, or CON rest periods). Arrows indicate the fixed assessment sequence at each time point (blood draw → VAS → Sargent jump → RAST). Dashed vertical lines denote the boundaries between the study days. 1RM, one-repetition maximum; CON, control; DOMS, delayed-onset muscle soreness; FR, foam rolling; RAST, Running-based Anaerobic Sprint Test; TENS, transcutaneous electrical nerve stimulation; VAS, visual analog scale

No adverse events were recorded during the intervention or blood collection sessions. Intervention compliance was 100%. The baseline characteristics did not differ among the groups (Table 1; all *p* > 0.40). The complete sequence and timing of familiarization, baseline testing, DOMS induction, recovery interventions, and follow-up assessments are presented in Fig. 2.

### Muscle soreness (Secondary outcome)

**Fig. 3:**
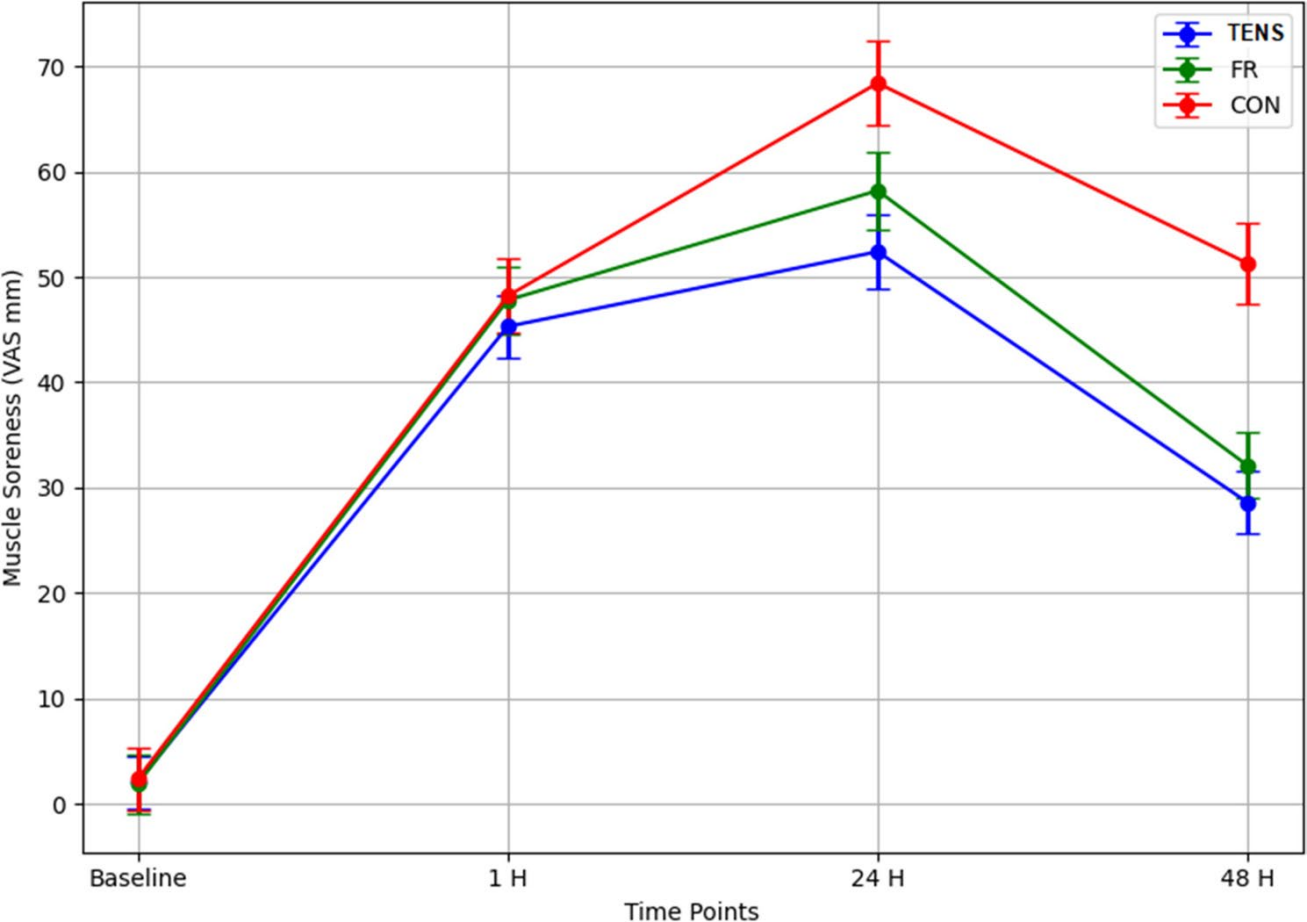
Changes in muscle soreness over time following transcutaneous electrical nerve stimulation, foam rolling, and control conditions. Caption: Muscle soreness was assessed using a 100-mm visual analog scale (VAS) at baseline, 1, 24, and 48 h postexercise in the TENS, FR, and CON groups. Both the TENS and FR groups demonstrated significantly reduced soreness compared with the CON group at 48 h. Error bars represent the standard deviation (*p* < 0.05). Legend: Each line represents the mean VAS score for one group across time points. Error bars indicate the standard deviation. Asterisks denote statistically significant between-group differences at 48 h (*p* < 0.05)

The eccentric exercise protocol induced DOMS in all three groups of participants. The VAS scores increased from near-zero baseline values to peak values at 24 h, reaching 52–68 mm across groups (Table 2). No significant between-group differences were observed at baseline or 1 h postexercise (*p* > 0.05). A significant Group × Time interaction was observed (F₍₆,₈₁₎ = 7.83, *p* < 0.001, η²*p* = 0.22). At 24 h, the TENS group reported significantly lower soreness than the CON group (mean difference − 16.0 mm, 95% CI: −29.8, − 2.2; *p* = 0.024, d = 1.10). By 48 h, both intervention groups reported significantly lower VAS scores than the CON group (TENS vs. CON: −22.7 mm, 95% CI: −34.8, − 10.6; *p* < 0.001, d = 1.78; FR vs. CON: −19.2 mm, 95% CI: −31.3, − 7.1; *p* = 0.003, d = 1.48). No significant differences were observed between the TENS and FR groups at any time point (all *p* > 0.40). The VAS data for muscle soreness at each time point are presented in Table 2, and the changes over time are shown in Fig. 3. Because the participants were aware of their group allocation, VAS-rated soreness was susceptible to expectancy or placebo effects. This vulnerability is inherent to unblinded designs involving perceptible interventions and should be considered when interpreting the subjective outcomes.

**Table 2:** Muscle soreness (visual analog scale) with pairwise comparisons

Time Point	TENS (mm)	FR (mm)	CON (mm)	Comparison	Mean Difference [95% CI]	*p*-value	Cohen’s d
Baseline	2.1 ± 1.8	1.9 ± 2.0	2.4 ± 1.6	TENS vs. CON	-0.3 [-2.0, 1.4]	0.724	0.18
				FR vs. CON	-0.5 [-2.2, 1.2]	0.562	0.28
				TENS vs. FR	0.2 [-1.5, 1.9]	0.816	0.11
1 h	45.3 ± 12.1	47.8 ± 11.4	48.2 ± 13.2	TENS vs. CON	-2.9 [-12.8, 7.0]	0.562	0.23
				FR vs. CON	-0.4 [-10.3, 9.5]	0.936	0.03
				TENS vs. FR	-2.5 [-12.4, 7.4]	0.618	0.21
24 h	52.4 ± 14.2	58.2 ± 13.8	68.4 ± 15.1	**TENS vs. CON**	**-16.0 [-29.8**,** -2.2]**	**0.024**	**1.10**
				FR vs. CON	-10.2 [-24.0, 3.6]	0.146	0.71
				TENS vs. FR	-5.8 [-19.6, 8.0]	0.408	0.42
48 h	28.6 ± 10.4	32.1 ± 11.2	51.3 ± 14.8	**TENS vs. CON**	**-22.7 [-34.8**,** -10.6]**	**< 0.001**	**1.78**
				**FR vs. CON**	**-19.2 [-31.3**,** -7.1]**	**0.003**	**1.48**
				TENS vs. FR	-3.5 [-15.6, 8.6]	0.568	0.33

Values are presented as mean ± standard deviation. Omnibus Group × Time interaction: F_(6,81)_ = 7.83, *p* < 0.001, η²*p* = 0.22. All pairwise comparisons were derived from estimated marginal means using the pooled error term from the mixed-design repeated-measures ANOVA. Bonferroni adjustment was applied for multiple comparisons at each time point. Bold indicates statistical significance at *p* < 0.05

*CI* Confidence interval, *CON* Control group, *FR* Foam rolling, *TENS* Transcutaneous electrical nerve stimulation, *VAS* Visual analog scale

### CK responses

**Fig. 4:**
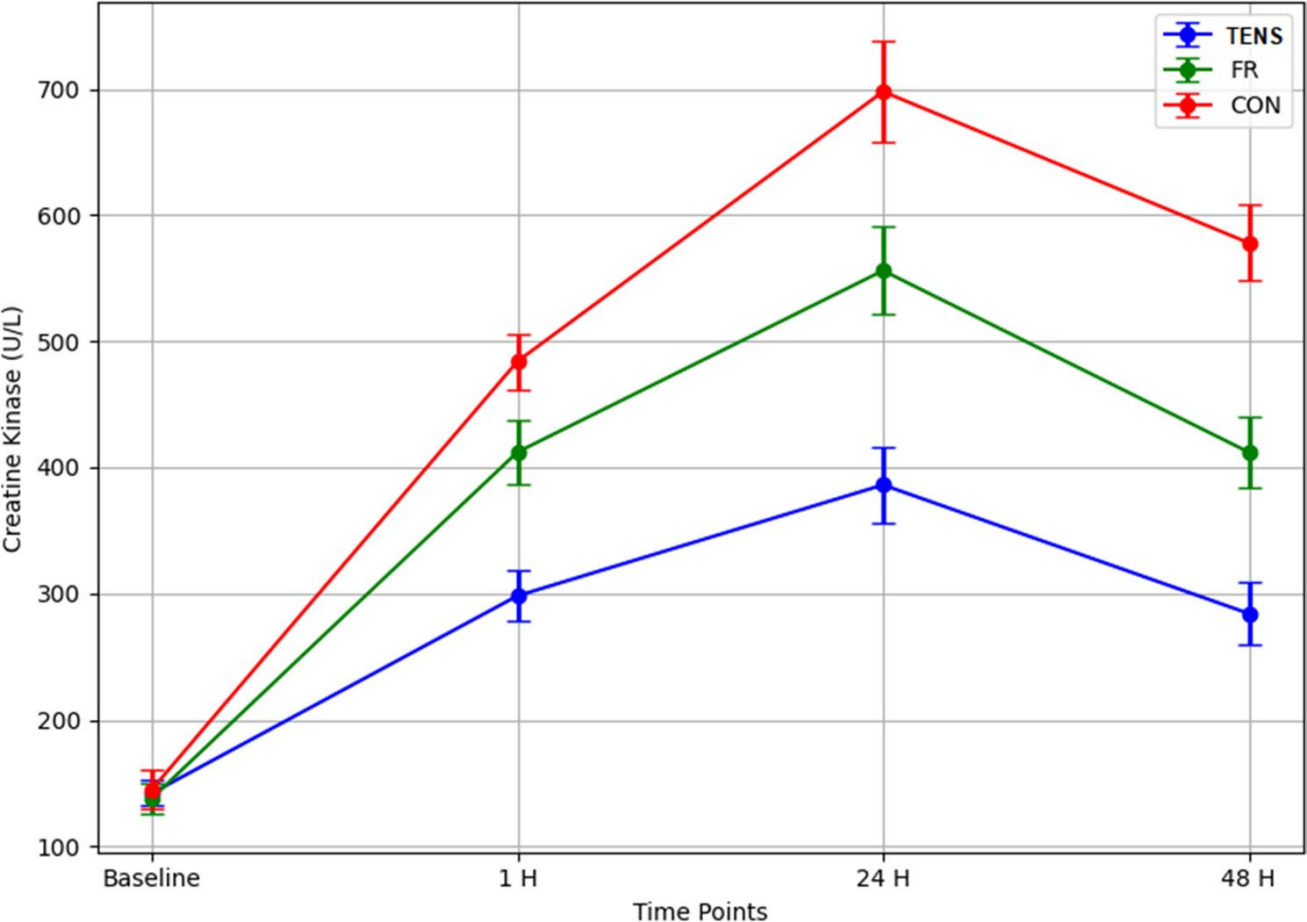
Comparison of serum creatine kinase concentrations across time in response to transcutaneous electrical nerve stimulation, foam rolling, and control conditions. Caption: Serum CK concentrations at baseline and at 1, 24, and 48 h following eccentric exercise are presented for the TENS, FR, and CON groups. The TENS group exhibited significantly lower CK concentrations than the CON group at all postexercise time points, and The FR group exhibited significantly lower CK concentrations than the CON group at 48 h. Error bars represent standard deviation (*p* < 0.05). Legend: Each line represents the mean serum CK concentration for one group across time points. Error bars indicate standard deviation. Asterisks denote statistically significant between-group differences at relevant time points (*p* < 0.05)

Baseline CK concentrations were comparable across the groups (TENS: 142 ± 38 U/L; FR: 138 ± 41 U/L; CON: 145 ± 35 U/L; *p* = 0.912). The eccentric protocol produced marked elevations in all groups (Fig. 4; Table 3). The serum CK concentrations at baseline and 1, 24, and 48 h postexercise are presented in Table 3.

**Table 3:** Serum creatine kinase concentrations with pairwise comparisons

Time Point	TENS (U/L)	FR (U/L)	CON (U/L)	Comparison	Mean Difference [95% CI]	*p*-value	Cohen’s d
Baseline	142 ± 38	138 ± 41	145 ± 35	TENS vs. CON	−3 [− 38, 32]	0.864	0.08
				FR vs. CON	−7 [− 43, 29]	0.701	0.18
				TENS vs. FR	4 [− 31, 39]	0.821	0.10
1 h	298 ± 72	412 ± 98	484 ± 112	TENS vs. CON	−186 [− 289, − 83]	**0.001**	**1.98**
				FR vs. CON	−72 [− 175, 31]	0.184	**0.68**
				TENS vs. FR	−114 [− 217, − 11]	**0.031**	**1.33**
24 h	386 ± 94	556 ± 134	698 ± 165	TENS vs. CON	−312 [− 442, − 182]	**0.001**	**2.32**
				FR vs. CON	−142 [− 272, 12]	0.078	**0.94**
				TENS vs. FR	−170 [− 300, − 40]	**0.012**	**1.47**
48 h	284 ± 68	412 ± 108	578 ± 142	TENS vs. CON	−294 [− 401, − 187]	**0.001**	**2.64**
				FR vs. CON	−166 [− 273, − 59]	**0.003**	**1.32**
				TENS vs. FR	−128 [− 235, − 21]	**0.024**	**1.42**

Values are presented as mean ± standard deviation expressed in units per liter. Omnibus Group × Time interaction: F_(6,81)_ = 8.42, *p* < 0.001, η²*p* = 0.24. All pairwise comparisons were derived from estimated marginal means using the pooled error term from the mixed-design repeated-measures ANOVA. Bonferroni adjustment was applied for multiple comparisons at each time point. Bold indicates statistical significance at *p* < 0.05

*CI* Confidence interval, *CON* Control group, *FR* Foam rolling, *TENS* Transcutaneous electrical nerve stimulation, *U/L* Units per liter

A significant Group × Time interaction was observed (F₍₆,₈₁₎ = 8.42, *p* < 0.001, η²*p* = 0.24). Follow-up analyses yielded the following results.

At 1 h, CK concentration was significantly lower in the TENS group than in the CON group (mean difference − 186 U/L, 95% CI: −289, − 83; *p* = 0.001, d = 1.98). No significant difference was observed between the FR and CON groups (*P* = 0.184). CK concentrations over time are shown in Fig. 4. Given the typically delayed time course of CK release following eccentric exercise, this early between-group difference should be interpreted with the caveats discussed below.

At 24 h, CK concentrations were lower in both intervention groups than in the CON group, although only the TENS group reached statistical significance (TENS vs. CON: mean difference, − 312 U/L; *p* = 0.001, d = 2.32; FR vs. CON: mean difference, − 142 U/L; *p* = 0.078, d = 0.94).

At 48 h, both intervention groups had significantly lower CK concentrations than the CON group (TENS vs. CON: *p* = 0.001, d = 2.64; FR vs. CON: *p* = 0.003, d = 1.32). The TENS group also had significantly lower CK concentration than the FR group (*p* = 0.024, d = 1.42).

### Vertical jump performance

Baseline jump heights were equivalent across the groups (TENS: 42.8 ± 4.2 cm; FR: 41.9 ± 3.8 cm; CON: 42.4 ± 4.5 cm; *p* = 0.874). Jump performance declined in all groups at 1 and 24 h postexercise. Vertical jump height data at different time points are presented in Table 4.

**Table 4:** Vertical jump height recovery with pairwise comparisons

Time Point	TENS (cm)	FR (cm)	CON (cm)	Comparison	Mean Difference [95% CI]	*p*-value	Cohen’s d
Baseline	42.8 ± 4.2	41.9 ± 3.8	42.4 ± 4.5	TENS vs. CON	0.4 [-3.8, 4.6]	0.851	0.09
				FR vs. CON	-0.5 [-4.6, 3.6]	0.810	0.11
				TENS vs. FR	0.9 [-3.2, 5.0]	0.664	0.15
1 h	38.4 ± 3.9	37.8 ± 4.2	37.2 ± 4.1	TENS vs. CON	1.2 [-2.1, 4.5]	0.472	0.30
				FR vs. CON	0.6 [-2.7, 3.9]	0.718	0.15
				TENS vs. FR	0.6 [-2.7, 3.9]	0.718	0.15
24 h	36.2 ± 4.1	35.4 ± 3.7	34.8 ± 4.4	TENS vs. CON	1.4 [-1.9, 4.7]	0.402	0.33
				FR vs. CON	0.6 [-2.7, 3.9]	0.718	0.15
				TENS vs. FR	0.8 [-2.5, 4.1]	0.634	0.20
48 h	41.2 ± 3.8	40.1 ± 4.1	36.4 ± 4.8	**TENS vs. CON**	**4.8 [2.1**,** 7.5]**	**0.001**	**1.11**
				**FR vs. CON**	**3.7 [1.4**,** 6.4]**	**0.001**	**0.85**
				TENS vs. FR	1.1 [-1.4, 3.6]	0.312	0.28

Values are presented as mean ± standard deviation expressed in centimeters. Omnibus Group × Time interaction: F_(6,81)_ = 4.89, *p* = 0.008, η²*p* = 0.15. All pairwise comparisons were derived from estimated marginal means using the pooled error term from the mixed-design repeated-measures ANOVA. Bonferroni adjustment was applied for multiple comparisons at each time point. Bold indicates statistical significance at *p* < 0.05

*CI* Confidence interval, *CON* Control group, *FR* Foam rolling, *TENS* Transcutaneous electrical nerve stimulation

A significant Group × Time interaction was observed (F₍₆,₈₁₎ = 4.89, *p* = 0.008, η²*p* = 0.15). By 48 h, both intervention groups showed a clear recovery advantage in vertical jump performance compared with passive rest, indicating that active recovery accelerated the return of explosive function (Table 4).

- TENS vs. CON: +4.8 cm (95% CI: 2.1, 7.5), *p* = 0.001, d = 1.11
- FR vs. CON: +3.7 cm (95% CI: 1.4, 6.4), *p* = 0.001, d = 0.85
- No significant difference was observed between the TENS and FR groups (*p* = 0.312)

**Fig. 5:**
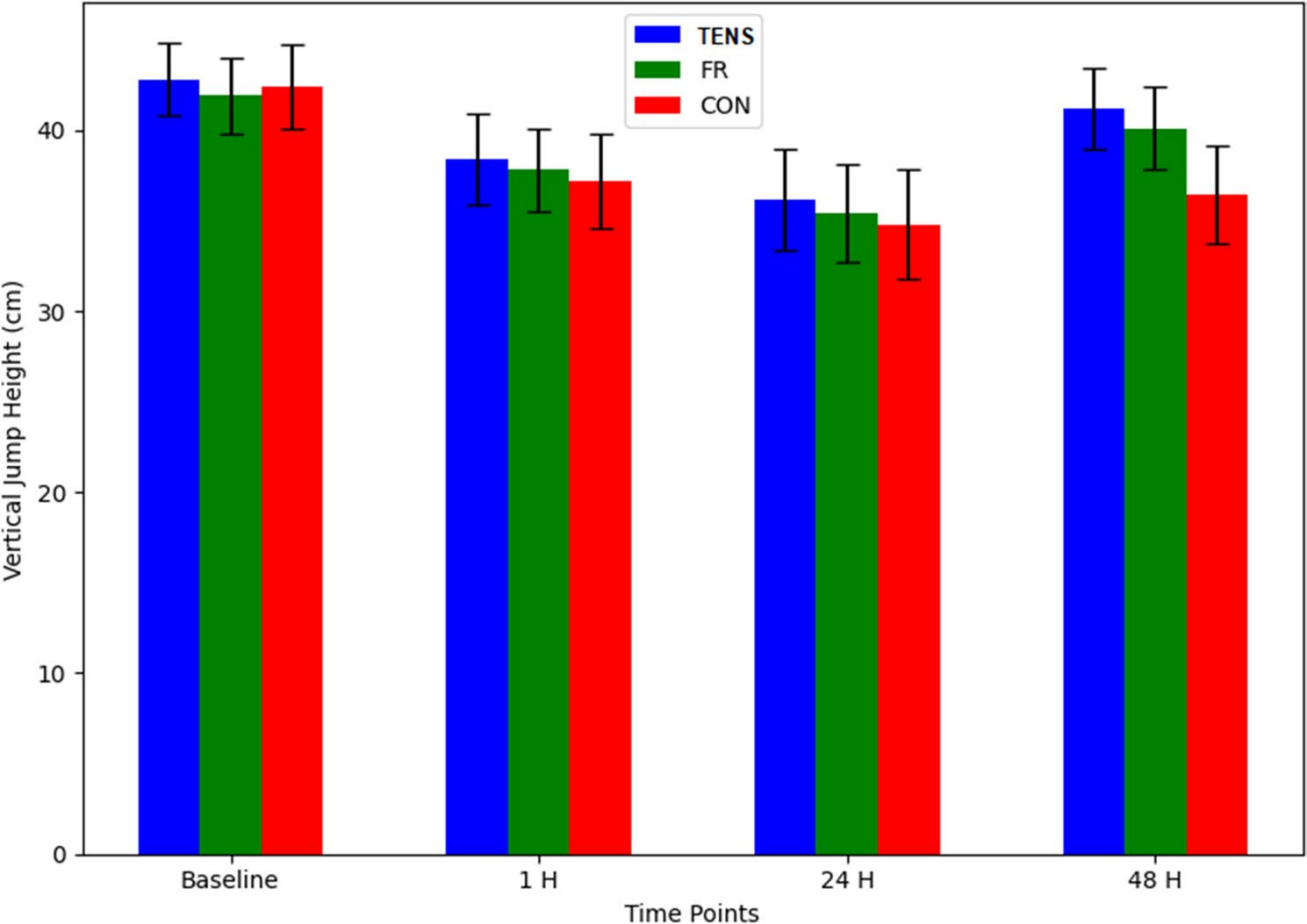
Vertical jump performance recovery at 1, 24, and 48 h following transcutaneous electrical nerve stimulation, foam rolling, and control conditions. Caption: Vertical jump height (cm) at baseline, 1, 24, and 48 h postexercise for the TENS, FR, and CON groups. The TENS and FR groups demonstrated significantly greater recovery of vertical jump height than the CON group at 48 h (*p* < 0.05). Error bars represent the standard deviation. Legend: Bar heights represent the mean vertical jump height for each group at each time point. Error bars indicate the standard deviation. Asterisks denote statistically significant between-group differences at 48 h (*p* < 0.05)

At 48 h, jump height recovered to 96.3% of baseline in the TENS group (41.2 ± 3.8 cm) and 95.7% in the FR group (40.1 ± 4.1 cm), compared with 85.8% in the CON group (36.4 ± 4.8 cm). Vertical jump performance across all groups is shown in Fig. 5.

### Anaerobic power

Analysis of RAST-derived peak power revealed no significant Group × Time interaction (F₍₆,₈₁₎ = 2.34, *p* = 0.086, η²*p* = 0.08) and no significant main effect of Group (F₍₂,₂₇₎ = 1.92, *p* = 0.167, η²*p* = 0.06). Anaerobic peak power data are presented in Table 5.

**Table 5:** Anaerobic peak power with pairwise comparisons

Time Point	TENS (W/kg)	FR (W/kg)	CON (W/kg)	Comparison	Mean Difference [95% CI]	*p*-value	Cohen’s d
Baseline	11.2 ± 1.4	10.9 ± 1.3	11.1 ± 1.5	TENS vs. CON	0.1 [-0.9, 1.1]	0.892	0.07
				FR vs. CON	-0.2 [-1.2, 0.8]	0.784	0.14
				TENS vs. FR	0.3 [-0.7, 1.3]	0.624	0.22
1 h	9.8 ± 1.2	9.6 ± 1.4	9.4 ± 1.3	TENS vs. CON	0.4 [-0.7, 1.5]	0.784	0.32
				FR vs. CON	0.2 [-0.9, 1.3]	0.891	0.15
				TENS vs. FR	0.2 [-0.9, 1.3]	0.891	0.15
24 h	10.4 ± 1.3	10.2 ± 1.2	9.5 ± 1.4	TENS vs. CON	0.9 [-0.2, 2.0]	0.089	0.61
				FR vs. CON	0.7 [-0.4, 1.8]	0.142	0.54
				TENS vs. FR	0.2 [-0.9, 1.3]	0.891	0.16
48 h	10.8 ± 1.2	10.6 ± 1.3	10.1 ± 1.5	TENS vs. CON	0.7 [-0.4, 1.8]	0.324	0.52
				FR vs. CON	0.5 [-0.6, 1.6]	0.412	0.38
				TENS vs. FR	0.2 [-0.9, 1.3]	0.891	0.16

Values are presented as mean ± standard deviation expressed in watts per kilogram. Omnibus Group × Time interaction: F_(6,81)_ = 2.34, *p* = 0.086, η²*p* = 0.08. Main effect of group: F(2,27) = 1.92, *p* = 0.167, η²*p* = 0.06. All pairwise comparisons were derived from estimated marginal means using the pooled error term from the mixed-design repeated-measures ANOVA. Bonferroni adjustment was applied for multiple comparisons at each time point. Bold indicates statistical significance at *p* < 0.05

*CI* Confidence interval, *CON* Control group, *FR* Foam rolling, *TENS* Transcutaneous electrical nerve stimulation, *W/kg* Watts per kilogram

At 24 h, medium effect sizes were observed in both intervention groups compared with the CON group (TENS vs. CON: d = 0.61, *p* = 0.089; FR vs. CON: d = 0.54, *p* = 0.142). At 48 h, effect sizes were smaller (TENS vs. CON: d = 0.52; FR vs. CON: d = 0.38), with no significant differences (*p* > 0.20). Similar patterns were observed for mean power and fatigue index, with no statistically significant effects at any time point (see Supplementary Table S1 for details). Sensitivity analysis indicated adequate statistical power (81%) to detect effects of the magnitude observed for the Group × Time interaction. This suggests, but does not guarantee, that the null finding reflects a genuine absence of meaningful group differences, rather than a Type II error. The modest sample size and metabolic specificity of repeated sprint performance, which depends heavily on phosphocreatine resynthesis and glycolytic capacity, may both contribute to the absence of statistically detectable effects within the 48-h window examined.

## Discussion

To our knowledge, this study is among the first to compare the effects of TENS and FR as postexercise recovery strategies following eccentric exercise–induced muscle damage in elite female volleyball players. Both active modalities accelerated functional recovery and reduced indirect markers of muscle damage relative to passive rest, but they did so through distinct temporal pathways. TENS produced earlier biochemical effects, with significantly lower CK concentrations evident within 1 h postexercise, whereas FR achieved comparable functional restoration, measured by vertical jump performance, only after 48 h. This dissociation between biochemical and functional recovery timelines has direct practical implications for recovery modality selection in high-performance settings. However, given the specific biochemical markers employed and the relatively small sample size, this investigation should be viewed as an exploratory pilot study intended to generate hypotheses for larger-scale clinical trials.

### Effects on muscle damage and soreness

The most notable finding was the difference in recovery timing between the interventions, with TENS and FR influencing biochemical markers along distinct trajectories rather than producing uniform effects. Lower CK concentrations were observed in the TENS group 1 h postexercise. However, this finding warrants cautious interpretation. CK typically follows a delayed release trajectory after eccentric exercise, with peak concentrations between 24 and 96 h [5]. The short interval between stimulation completion and blood sampling (approximately 10 min) raises questions about whether a genuine intervention effect on CK kinetics could manifest within this window. The early between-group separation may reflect the modulation of ongoing protein efflux from already damaged fibers, interindividual biological variability amplified by small group sizes, or stochastic sampling effects rather than accelerated systemic clearance. This early separation persisted for 48 h, with TENS consistently maintaining the lowest concentration among the three groups.

The 3.3-fold CK elevation at 1 h in the CON group was earlier than the 2–6 h onset typically reported after eccentric exercise [5]. This rapid rise likely reflects the high exercise volume (5 sets × 15 repetitions at 110% 1RM) combined with the training status of the participants; elite athletes produce greater absolute eccentric forces, which may accelerate initial membrane disruption and protein release [4]. Nevertheless, the mechanisms underlying the lower CK concentrations in the TENS group at 1 h require careful consideration. The short interval between stimulation and blood sampling (approximately 10 min) may be insufficient for measurable changes in CK clearance, given the approximately 1.5-day serum half-life of this enzyme [5]. An alternative explanation is that high-frequency TENS attenuates further CK release from already damaged fibers rather than accelerating systemic clearance. Previous laboratory work suggests that high-frequency TENS may enhance local microcirculation through vasodilatory and subthreshold contractile mechanisms [37]. Whether such effects contributed to the CK patterns observed in the present study cannot be established from the available data, as neither tissue perfusion nor sarcolemmal integrity was directly assessed. Lymphatic flow modification and reduced interstitial mediator accumulation have been proposed as additional TENS-related mechanisms [12]; however, these pathways were not measured in the present study, and their contribution to the observed CK differences remains speculative.

FR did not differ significantly from that of the CON group at 24 h (*p* = 0.078). This comparison was statistically significant only at 48 h. The observed trajectory is consistent with a delayed response, although the nonsignificant 24-h finding precludes firm conclusions regarding intermediate time points. FR primarily affects superficial tissues through direct mechanical compression, improving fascial mobility, and reducing muscle stiffness without immediately influencing deep tissue perfusion to the same extent as electrical modalities [38]. The delayed CK response in the FR group is consistent with proposed mechanisms involving progressive improvements in tissue compliance and fascial viscosity [10, 38], although these remain hypothetical in the absence of direct tissue-level measurements. Autonomic modulation has also been proposed as a contributing pathway [10], but it was not assessed in this investigation.

The CK effect sizes observed in the present study (TENS vs. CON: d = 1.98–2.64 across 1–48 h) substantially exceeded the pooled effect sizes for electrical modalities reported in a previous meta-analysis (approximately 0.40–0.80) [6]. Several factors may account for the discrepancy between the present effect sizes and prior pooled estimates. First, most meta-analytic datasets aggregate recreational and mixed-sex cohorts, whose lower absolute eccentric force production generates smaller CK elevations, thereby compressing between-group separation. Second, the high eccentric volume used here (five sets of 15 repetitions at 110% 1RM) exceeds the protocols typical of many included studies, amplifying the signal available for intervention effects. Third, small samples inflate standardized effect sizes, and the present estimates should be interpreted with this caveat. The convergence of these factors, not any single explanation, likely underlies the large observed effects. Malone et al. [12] reported CK reductions of approximately 20–35% following neuromuscular electrical stimulation after eccentric exercise, whereas the present TENS group showed reductions of 45–51% relative to the CON group at 24–48 h postexercise. The inconsistency in FR-related CK findings across the literature deserves scrutiny. Pearcey et al. [26] reported significant CK reductions in recreationally active men using a three-bout FR protocol, whereas D’Amico and Gillis [27] found no effect using a single-session design. Skinner et al. [19] systematically catalogued sources of heterogeneity — including rolling duration, applied pressure, timing relative to exercise, and target muscle groups — and concluded that protocol standardization remains a critical barrier to cross-study comparison. The present protocol (three cycles of five bilateral regions at a standardized cadence) represents one configuration among many, and its specific contribution to the observed 48-h CK reduction cannot be isolated from other design elements. Notably, Zhang et al. [22] reported that FR reduced CK and lactate concentrations in elite volleyball athletes, although their protocol differed from that used in the present study in terms of rolling duration and target muscle groups. A follow-up study by the same group [23] using thermal imaging confirmed that FR altered local tissue perfusion patterns, lending mechanistic support to the delayed biochemical benefits observed in the present investigation.

Both intervention groups also reported significantly lower muscle soreness than the CON group at 48 h, with large effect sizes. Soreness reduction did not differ between the TENS and FR groups at any time point. This parallel trajectory in subjective pain relief, despite divergent CK timelines, reinforces the view that perceived soreness and structural membrane damage are partially independent constructs [1, 3]. Both modalities may reduce pain perception through overlapping sensory mechanisms: TENS through preferential activation of large-diameter afferents and gate control modulation [8, 9], and FR through pressure-mediated stimulation of mechanoreceptors and Golgi tendon organs [10, 15].

### Functional performance recovery

Vertical jump performance serves as an ecologically valid recovery indicator in volleyball, where a single match can involve more than 100 maximal jumps [20]. Jump height improved in all three groups between 24 and 48 h, consistent with the expected natural recovery trajectory following eccentric exercise. However, the residual deficit at 48 h was substantially larger in the CON group (85.8% of baseline) than in either intervention group (TENS: 96.3%; FR: 95.7%), suggesting that both active modalities accelerated the rate of recovery rather than preventing initial impairment (Table 4). The design does not permit complete separation of intervention-specific effects from spontaneous recovery, and the magnitude of added benefit attributable to each modality — beyond what would have occurred with rest alone — remains uncertain. In a sport where success often depends on centimeters of vertical displacement, this 10-percentage-point recovery gap is of direct competitive relevance [20].

The observation that the TENS and FR groups achieved comparable jump recovery, despite differing biochemical timelines, reinforces the finding that functional restoration does not map directly onto circulating damage markers. Our data demonstrate a clear dissociation between biochemical trajectories and functional recovery. It is critical to acknowledge that CK serves primarily as a circulating marker of sarcolemmal permeability and muscle membrane disruption, rather than a direct indicator of contractile capacity or total functional readiness — a concern echoed by Owens et al. [4], who argued that circulating proteins reflect membrane permeability rather than contractile capacity per se. Therefore, its isolated interpretation as a standalone recovery marker remains constrained. Soreness, neuromuscular fatigue, and psychological readiness each contribute to performance decrements through pathways that operate largely independently of circulating enzyme concentrations [3, 4]. Performance restoration likely depends on the resolution of multiple parallel processes, including pain inhibition, neuromuscular re-recruitment, and psychological readiness. Consequently, practitioners should prioritize functional performance metrics over isolated biochemical indices when evaluating an athlete’s readiness to return to train or compete.

The dissociation between CK trajectories and vertical jump recovery has broader implications for applied monitoring. If circulating CK does not reliably predict functional readiness, its standalone value as a recovery biomarker diminishes in sports settings, where performance capacity is the primary concern. Owens et al. [4] have argued that circulating intracellular proteins reflect sarcolemmal permeability rather than contractile function per se — a distinction that the present data reinforce. Therefore, decision-making should prioritize functional testing over isolated biochemical markers when evaluating an athlete’s readiness to train or compete.

The null findings for the RAST-derived anaerobic power require a different explanation. RAST demands six consecutive maximal sprints separated by only 10-s rest intervals, placing high demands on phosphocreatine resynthesis and glycolytic capacity, which may be less responsive to the peripheral mechanisms targeted by TENS and FR than the neuromuscular determinants of single-effort jumping. The medium effect sizes at 24 h (d = 0.54–0.61) suggest that a real but modest effect may exist; however, sensitivity analysis confirmed adequate power (81%) to detect the observed interaction, indicating that these null findings do not simply reflect a small sample size. Alternatively, anaerobic sprint capacity may require longer recovery periods than the 48-h window examined in this study [6].

### Clinical and practical implications

These findings have several practical implications for practitioners working with volleyball athletes and, more broadly, with athletes whose sports impose heavy eccentric lower limb demands [21].

First, even when applied unilaterally in this study, TENS may offer advantages when short-term biochemical attenuation is desired, for example, in tournament settings with consecutive-day competitions. However, this potential advantage was limited to CK concentrations and did not consistently extend to functional or subjective outcomes. Both modalities yielded equivalent jump recovery and soreness reduction by 48 h, and neither demonstrated clear superiority across all the measured domains. Practitioners should consider the logistical requirements of each modality (TENS requires trained supervision and equipment, whereas FR is self-administered and portable) in conjunction with these outcome-specific findings when making recovery decisions. The benefits observed as early as 1 h after exercise suggest that TENS can be incorporated into recovery protocols immediately after competition. A clinical-grade- TENS unit costs approximately $50–150 and requires trained supervision, which may limit its use in resource-constrained- environments.

Second, FR remains a practical and effective alternative, offering comparable functional restoration at 48 h at a fraction of the cost ($10–30 for a standard roller) and with the advantage of self-administration without the need for specialized personnel [15, 16, 19]. Although the effects on CK were less pronounced than those of TENS, the equivalent jump recovery suggests that FR adequately addresses the functional limitations most relevant to on-court performance [15, 26].

Third, individual variation in CK response was substantial (standard deviations of 68–165 U/L at postexercise time points), suggesting that a uniform recovery regimen may be suboptimal. Practitioners should monitor individual recovery trajectories through either biomarker sampling or standardized performance testing when selecting between modalities.

Fourth, combining both modalities in sequence — immediate postexercise TENS followed by FR during subsequent recovery periods — could leverage the distinct mechanisms of each intervention [6, 11, 38]. Although this hypothesis was not tested in the present study, it warrants direct investigation, and network meta-analytic evidence supports the notion that combining modalities may yield superior outcomes [39].

### Limitations and future directions

Several design features should be considered when interpreting these findings. The relatively small sample size (*n* = 10 per group) limits the precision of effect estimates, increases susceptibility to sampling variability, and means that the very large effect sizes observed for CK (d = 1.98–2.64) may be inflated — particularly given the high biological variability of this enzyme. These estimates should therefore be treated as preliminary rather than definitive population parameters, and the findings should be interpreted accordingly.

First, although randomization and allocation concealment were implemented, participant blinding was not feasible because of the nature of the interventions. Although primary biochemical and performance outcomes are less susceptible to expectancy bias than self-reported measures, the significant VAS differences observed between the groups may partly reflect awareness of receiving an active intervention rather than a purely physiological effect. To further minimize bias, we implemented concealed allocation, verified baseline equivalence, standardized all procedures, and ensured a blinded outcome assessment. Nonetheless, some residual confounding, particularly for subjective endpoints, cannot be entirely excluded.

Second, TENS was applied unilaterally to the dominant leg, whereas the eccentric protocol and all functional assessments were bilateral; this mismatch yielded a conservative estimate of TENS efficacy but complicated direct comparison with the bilaterally administered FR protocol. The applied pressure during foam rolling was not objectively measured, and differences in bodyweight distribution may have affected dose consistency across participants, a limitation common to most FR studies but one that restricts confidence in dose–response inferences.

Third, the menstrual cycle phase was neither standardized nor recorded, and hormonal fluctuations can influence CK release and clearance, which is an important consideration for future female-specific studies [22, 23].

Fourth, the sample consisted exclusively of elite female volleyball players, limiting generalizability to male athletes, recreational populations, and other sports [2, 17]; recovery responses may be shaped by volleyball-specific demands in ways that do not directly transfer to other contexts [20, 21].

Fifth, the 48-h follow-up captured the typical DOMS peak but may have missed later divergences in the recovery trajectories [1, 3].

Sixth, only a single exercise bout was examined [31, 40]; the repeated bout effect may modify responsiveness to these interventions across successive training sessions, and future studies should evaluate the sustained effects over multiple microcycles [6, 19].

Seventh, creatine kinase was the sole biochemical marker of muscle damage. CK exhibits substantial inter-individual variability and does not consistently correlate with functional impairment or perceived muscle soreness. The absence of complementary biochemical markers (e.g., interleukin-6, tumor necrosis factor-α, myoglobin) and imaging-based indices of structural damage restricts the mechanistic conclusions that can be drawn from our data. Importantly, the lack of direct neuromuscular or mechanistic assessments, such as maximal voluntary isometric contraction (MVIC) or electromyographic (EMG) analysis, prevents us from distinguishing between central and peripheral fatigue components. Future studies should incorporate these measures alongside ultrasound-based structural analysis to provide a more comprehensive view of the recovery mechanisms at play. Additional mechanistic outcomes, including muscle perfusion imaging, were not measured, further limiting the ability to explain the observed differences between modalities [4, 9, 37].

Eighth, this trial was retrospectively registered (ClinicalTrials.gov NCT07438197, registered on 22/02/2026). Although the study protocol was prospectively approved by an institutional ethics committee (IR.USB.REC.1399.007) and all outcomes were predefined in the approved protocol prior to data collection, retrospective registration limits external verification of outcome prespecification and introduces potential concerns regarding the transparency of reporting. Prospective registration is strongly recommended for future studies.

Future research should address these gaps through fully randomized designs with larger, sport-diverse cohorts, dose–response investigations that systematically vary stimulation parameters and rolling durations, and combined-modality protocols that test whether sequential TENS and FR produce additive or synergistic effects. Sex-disaggregated analyses should be standardized in all such investigations [22, 23].

Taken together, these findings position TENS and FR as complementary tools in the practitioner’s recovery arsenal: TENS for rapid biochemical stabilization when time between competitions is scarce, and FR for accessible, self-directed recovery when equipment and supervision are limited.

## Conclusion

When examined under standardized eccentric loading, TENS and FR supported recovery through different temporal pathways, highlighting that biochemical and functional restoration do not necessarily progress in parallel. TENS attenuated circulating CK levels earlier and more markedly, whereas FR achieved equivalent functional recovery, as measured by vertical jump performance, within 48 h. This dissociation between biochemical and functional timelines has practical implications: TENS may be preferable when rapid biochemical recovery is needed between closely scheduled competitions, whereas FR offers a portable, self-administered alternative that delivers comparable performance restoration over a slightly longer window. However, these findings were derived from a single eccentric bout in one athletic population and require confirmation through fully randomized designs with larger, sport-diverse cohorts. Future studies should prioritize dose–response optimization, evaluate combined-modality sequencing, and monitor recovery across repeated training microcycles to determine whether the temporal advantages of TENS persist under real-world periodization demands.

## Supplementary Information

Supplementary Material 1: Supplementary Table S1. Mean power and fatigue index across time points.

Supplementary Material 2.

Supplementary Material 3: Supplementary Visual Abstract Effect of Transcutaneous Electrical Nerve Stimulation and Foam Rolling on Muscle Recovery Following Eccentric Exercise: A Comparison with Control

## Data Availability

The de-identified dataset and full study protocol are available from the corresponding author upon reasonable request.

## Abbreviations

1RM: One-repetition maximum
ANOVA: Analysis of variance
BMI: Body mass index
CI: Confidence interval
CK: Creatine kinase
CON: Control group
DOMS: Delayed-onset muscle soreness
FR: Foam rolling
h: Hour
ICC: Intraclass correlation coefficient
mA: Milliampere
mL: Milliliter
RAST: Running-based Anaerobic Sprint Test
SD: Standard deviation
TENS: Transcutaneous electrical nerve stimulation
U/L: Units per liter
VAS: Visual analog scale
W/kg: Watts per kilogram
η²p: Partial eta squared
µs: Microsecond
