# Supplementary material for: TENS versus foam rolling for recovery after eccentric exercise–induced muscle damage in elite female volleyball players: an exploratory randomized controlled trial
Source: BMC Sports Sci Med Rehabil. 2026 Jun 3;18:304. doi: 10.1186/s13102-026-01782-x (PMC13326353; doi:10.1186/s13102-026-01782-x)
Supplement: Supplementary file 1 — Supplementary Material 1: Supplementary Table S1. Mean power and fatigue index across time points. [file 13102_2026_1782_MOESM1_ESM.docx]

**SUPPLEMENTARY INFORMATION**

**Supplementary Table S1** Mean power and fatigue index across time points

| **Group** | **Time Point** | **Mean Power (W/kg)** | **Fatigue Index (%)** |
| --- | --- | --- | --- |
| **TENS** | Baseline | 8.4 ± 1.1 | 42.3 ± 8.1 |
|  | 1 h | 7.4 ± 0.9 | 47.8 ± 9.0 |
|  | 24 h | 7.8 ± 1.0 | 45.6 ± 8.5 |
|  | 48 h | 8.1 ± 0.9 | 43.8 ± 8.2 |
| **FR** | Baseline | 8.2 ± 1.0 | 43.1 ± 8.4 |
|  | 1 h | 7.2 ± 1.1 | 48.9 ± 9.3 |
|  | 24 h | 7.7 ± 0.9 | 46.8 ± 8.7 |
|  | 48 h | 8.0 ± 1.0 | 44.5 ± 8.5 |
| **CON** | Baseline | 8.3 ± 1.1 | 42.8 ± 8.2 |
|  | 1 h | 7.1 ± 1.0 | 50.2 ± 9.8 |
|  | 24 h | 7.1 ± 1.1 | 50.8 ± 9.8 |
|  | 48 h | 7.6 ± 1.1 | 47.6 ± 9.4 |

**Mean Power — Mixed-Design ANOVA:**

- Group × Time interaction: F(6,81) = 1.92, p = 0.108, η²p = 0.06
- Main effect — Time: F(3,81) = 19.84, p < 0.001, η²p = 0.42
- Main effect — Group: F(2,27) = 1.38, p = 0.268, η²p = 0.05

**Fatigue Index — Mixed ANOVA:**

- Group × Time interaction: F(6,81) = 1.81, p = 0.128, η²p = 0.05
- Main effect — Time: F(3,81) = 23.42, p < 0.001, η²p = 0.46
- Main effect — Group: F(2,27) = 1.48, p = 0.244, η²p = 0.04

**Pairwise Comparisons at 48 h:**

| **Variable** | **Comparison** | **Mean Difference [95% CI]** | **p-value** | **Cohen's d** |
| --- | --- | --- | --- | --- |
| **Mean Power** | TENS vs. CON | 0.5 [-0.4, 1.4] | 0.214 | 0.46 |
|  | FR vs. CON | 0.4 [-0.5, 1.3] | 0.342 | 0.38 |
|  | TENS vs. FR | 0.1 [-0.8, 1.0] | 0.824 | 0.11 |
| **Fatigue Index** | TENS vs. CON | -3.8 [-9.2, 1.6] | 0.168 | 0.48 |
|  | FR vs. CON | -3.1 [-8.5, 2.3] | 0.258 | 0.40 |
|  | TENS vs. FR | -0.7 [-6.1, 4.7] | 0.798 | 0.09 |

*Values are* *presented as mean ± standard deviation. Mean power represents the average power output across six 35‑m sprints. Fatigue index was calculated as [(peak power – minimum power) / peak power] × 100. All pairwise comparisons were derived from estimated marginal means using the pooled error term from the mixed-design repeated-measures ANOVA. Bonferroni adjustment was applied for multiple comparisons at each time point. No pairwise comparisons reached statistical significance (all p > 0.05). CI, confidence interval; CON, control group; TENS, transcutaneous electrical nerve stimulation; FR, foam rolling; W/kg, watts per kilogram; η²p, partial eta squared.*
