# Supplementary material for: TENS versus foam rolling for recovery after eccentric exercise–induced muscle damage in elite female volleyball players: an exploratory randomized controlled trial
Source: BMC Sports Sci Med Rehabil. 2026 Jun 3;18:304. doi: 10.1186/s13102-026-01782-x (PMC13326353; doi:10.1186/s13102-026-01782-x)
Supplement: Supplementary file 2 — Supplementary Material 2. [file 13102_2026_1782_MOESM2_ESM.pdf]

CONSORT 2010 checklist of information to include when reporting a randomised trial

Section/Topic	Item No	Checklist item	Reported on section(s) / page(s) equivalent
**Title and abstract**	1a	Identification as a randomized trial in the title	Title: "... A Randomized Controlled Trial"
	1b	Structured summary of trial design, methods, results, and conclusions (for specific guidance see CONSORT for abstracts)	Abstract (structured with Background, Methods, Results, Conclusions)
**Introduction**			
Background and objectives	2a	Scientific background and explanation of rationale	Introduction (paragraphs 1–4)
	2b	Specific objectives or hypotheses	Introduction (final paragraph: hypothesis stated)
**Methods**			
Trial design	3a	Description of trial design (such as parallel, factorial) including allocation ratio	Methods > Study Design: three-arm, parallel-group RCT, 1:1:1 ratio
	3b	Important changes to methods after trial commencement (such as eligibility criteria), with reasons	No important changes were made to methods after trial commencement.
Participants	4a	Eligibility criteria for participants	Methods > Participants (inclusion/exclusion criteria)
	4b	Settings and locations where the data were collected	Methods > Study Design (Zahedan Olympic Village, Iran); Participants (recruitment via coaches/staff)

Section/Topic	Item No	Checklist item	Reported on section(s) / page(s) equivalent
Interventions	5	The interventions for each group with sufficient details to allow replication, including how and when they were actually administered	Methods > Recovery Interventions (detailed protocols for TENS, FR, and CON; timing: 30 min post-exercise and repeated at 24 h)
Outcomes	6a	Completely defined pre-specified primary and secondary outcome measures, including how and when they were assessed	Methods > Outcome Measures (CK primary; vertical jump, RAST, VAS secondary; timing: baseline, 1/24/48 h)
	6b	Any changes to trial outcomes after the trial commenced, with reasons	No changes to trial outcomes after commencement.
Sample size	7a	How sample size was determined	Methods > Statistical Analysis (sensitivity analysis with G*Power; post-hoc justification)
	7b	When applicable, explanation of any interim analyses and stopping guidelines	Not applicable (no interim analyses)
Randomization: Sequence generation	8a	Method used to generate the random allocation sequence	Methods > Randomization and Group Allocation (computer-generated using Random Allocation Software v2.0)
	8b	Type of randomization(s); details of any restriction (such as blocking and block size)	Simple randomization (no blocking or restrictions).
Allocation concealment mechanism	9	Mechanism used to implement the random allocation sequence (such as sequentially numbered containers), describing any steps taken to conceal the sequence until interventions were assigned	Methods > Randomization and Group Allocation (sequentially numbered, opaque, sealed envelopes; prepared by independent researcher)

Section/Topic	Item No	Checklist item	Reported on section(s) / page(s) equivalent
Implementation	10	Who generated the random allocation sequence, who enrolled participants, and who assigned participants to interventions	Methods > Randomization (independent researcher generated/prepared; enrollment implied via team coaches/staff; assignment via envelopes post-baseline)
Blinding	11a	If done, who was blinded after assignment to interventions (for example, participants, care providers, those assessing outcomes) and how	Methods > Study Design (participant blinding not feasible; outcome assessors and data analysts blinded)
	11b	If relevant, description of the similarity of interventions	Not applicable (interventions dissimilar: TENS electrical, FR mechanical, CON rest)
Statistical methods	12a	Statistical methods used to compare groups for primary and secondary outcomes	Methods > Statistical Analysis (mixed-design RM ANOVA, Bonferroni post-hoc, effect sizes η^2p and d)
	12b	Methods for additional analyses, such as subgroup analyses and adjusted analyses	Not performed (no subgroups or adjusted analyses reported)
**Results**			
Participant flow (a diagram is strongly recommended)	13a	For each group, the numbers of participants who were randomly assigned, received intended treatment, and were analyzed for the primary outcome	Results (first paragraph); Fig. 1 CONSORT Flow Diagram (n=10 per group randomized, all completed, no losses)
	13b	For each group, losses and exclusions after randomization, together with reasons	Results (no losses/exclusions; 100% compliance)
Recruitment	14a	Dates defining the periods of recruitment and follow-up	Methods > Study Design (September–December 2020)

Section/Topic	Item No	Checklist item	Reported on section(s) / page(s) equivalent
	14b	Why the trial ended or was stopped	Trial completed as planned; no early stopping.
Baseline data	15	A table showing baseline demographic and clinical characteristics for each group	Table 1 (baseline characteristics; no differences, $p > 0.40$)
Numbers analysed	16	For each group, number of participants (denominator) included in each analysis and whether the analysis was by original assigned groups	Results (n=10 per group in all analyses; intention-to-treat implied as all completed)
Outcomes and estimation	17a	For each primary and secondary outcome, results for each group, and the estimated effect size and its precision (such as 95% confidence interval)	Results (Tables 2–5; Figs. 2–4; mean differences, 95% CIs, d, p-values reported for key comparisons)
	17b	For binary outcomes, presentation of both absolute and relative effect sizes is recommended	Not applicable (no binary outcomes)
Ancillary analyses	18	Results of any other analyses performed, including subgroup analyses and adjusted analyses, distinguishing pre-specified from exploratory	No subgroup, adjusted, or other exploratory analyses were performed.
Harms	19	All-important harms or unintended effects in each group (for specific guidance see CONSORT for harms)	Results (no adverse events recorded during interventions or blood collection)
**Discussion**			
Limitations	20	Trial limitations, addressing sources of potential bias, imprecision, and, if relevant, multiplicity of analyses	Discussion > Limitations and Future Directions (multiple limitations discussed: no participant blinding,

Section/Topic	Item No	Checklist item	Reported on section(s) / page(s) equivalent
			unilateral TENS, no menstrual cycle standardization, single bout, elite females only, etc.)
Generalizability	21	Generalizability (external validity, applicability) of the trial findings	Discussion > Limitations (limited to elite female volleyball players; volleyball-specific demands)
Interpretation	22	Interpretation consistent with results, balancing benefits and harms, and considering other relevant evidence	Discussion (full interpretation; practical implications; TENS earlier biochemical effect, FR practical alternative)
**Other information**			
Registration	23	Registration number and name of trial registry	Abstract (ClinicalTrials.gov NCT07438197, retrospectively registered 27 February 2026)
Protocol	24	Where the full trial protocol can be accessed, if available	Available from the corresponding author on request.
Funding	25	Sources of funding and other support (such as supply of drugs), role of funders	Declarations > Funding: No external funding received
