# Supplementary material for: TENS versus foam rolling for recovery after eccentric exercise–induced muscle damage in elite female volleyball players: an exploratory randomized controlled trial
Source: BMC Sports Sci Med Rehabil. 2026 Jun 3;18:304. doi: 10.1186/s13102-026-01782-x (PMC13326353; doi:10.1186/s13102-026-01782-x)
Supplement: Supplementary file 3 — Supplementary Material 3: Supplementary Visual Abstract Effect of Transcutaneous Electrical Nerve Stimulation and Foam Rolling on Muscle Recovery Following Eccentric Exercise: A Comparison with Control [file 13102_2026_1782_MOESM3_ESM.docx]

**Supplementary Visual Abstract**


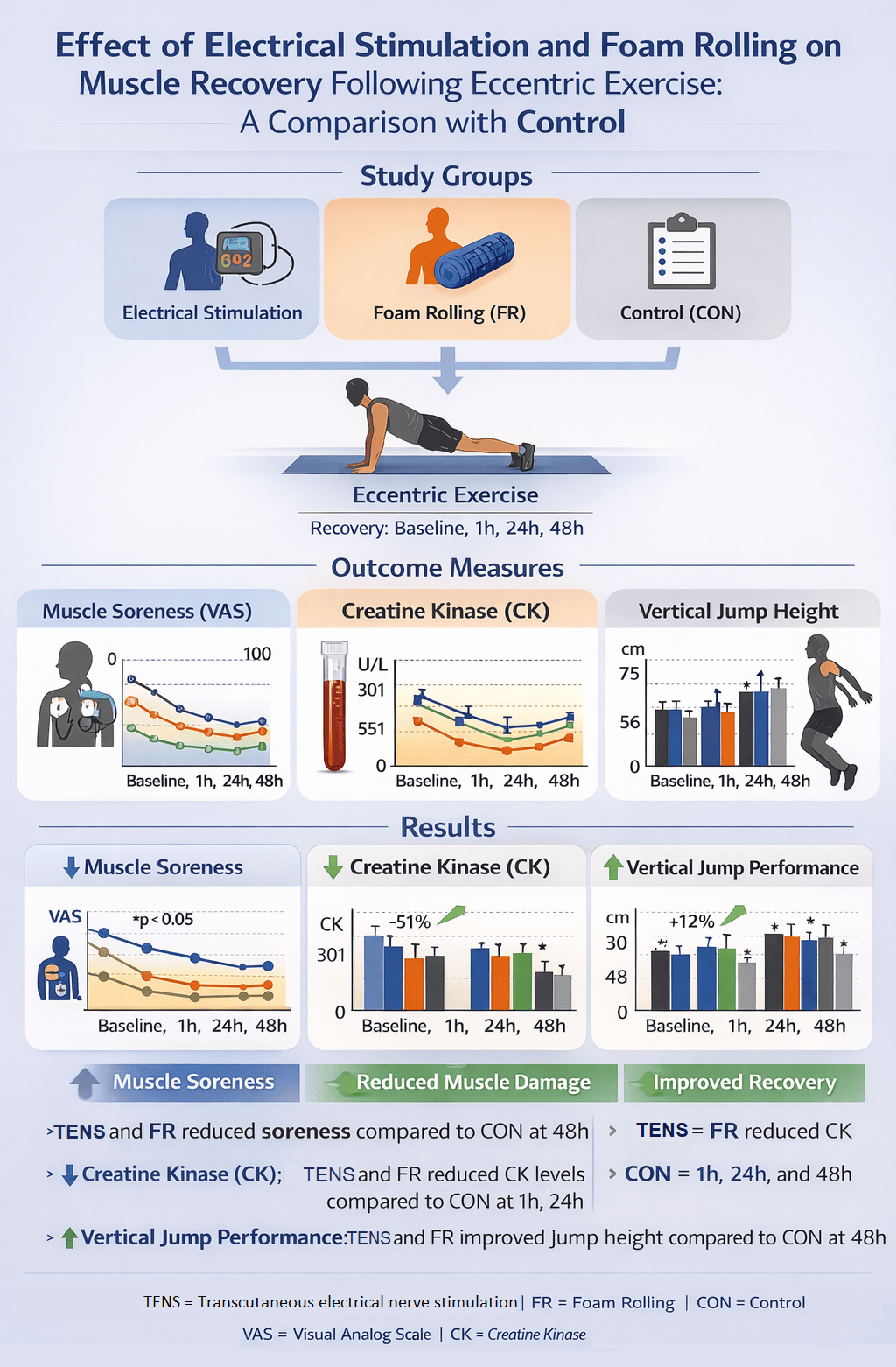


**Supplementary Visual Abstract** Effect of Transcutaneous Electrical Nerve Stimulation and Foam Rolling on Muscle Recovery Following Eccentric Exercise: A Comparison with Control

**Legend:** This visual abstract summarizes the effects of TENS, FR, and CON on muscle recovery following eccentric exercise. The key findings are as follows:

- Both TENS and FR reduced muscle soreness, as assessed using the VAS, and improved vertical jump performance compared with the CON condition.
- Serum CK concentrations, a biomarker of muscle damage, were significantly reduced in the TENS and FR groups than in the CON group.
- Significant improvements in vertical jump height recovery and muscle soreness were observed 48 h postexercise in both the TENS and FR groups.
- TENS demonstrated earlier and more pronounced effects on CK reduction, with significant differences observed as early as 1 h postexercise.
- FR provides a practical, self-administered recovery option with comparable functional outcomes at 48 h postexercise.
